# 36. Recurrent strokes in younger patient with rash: a case of Sneddon’s syndrome

**DOI:** 10.1093/rap/rky033.027

**Published:** 2018-09-20

**Authors:** Himashi Anver, Shirish Dubey, Siwalik Banerjee, Tanya Potter, Andrew Short, Andrew Ilchyshyn

**Affiliations:** 1 Rheumatology, University Hospital Coventry and Warwickshire, Coventry, UNITED KINGDOM; 2 Nephrology, University Hospital Coventry and Warwickshire, Coventry, UNITED KINGDOM; 3 Dermatology, University Hospital Coventry and Warwickshire, Coventry, UNITED KINGDOM

**Introduction:** Sneddon's syndrome, first described in 1965 is a rare clinical syndrome of multiple ischaemic strokes, neurological symptoms and livedo racemosa. It is thought to have an incidence of 4 per million and has an increased prevalence in females and younger adults. Our case is one of a patient with probable Sneddon’s syndrome, highlighting the diagnostic challenges and therapeutic dilemma.

**Case description:** This is a case of a 43 year old lady with a complex medical history, currently under the care of rheumatology, renal medicine, neurology and dermatology. She initially presented to the renal clinic with loin pain and haematuria. She had then been found to have a purpuric rash and multiple joint pain symptoms for which she as referred to rheumatology in 2005. In light of the haematuria, she underwent a renal biopsy and was found to have IgA nephropathy consistent with a diagnosis of Henoch Schonlein purpura. Her other co-morbidities included a previous ventricular septal defect, repaired in childhood and recurrent migraines. Following diagnosis, she was treated with oral glucocorticoids and azathioprine. Her symptoms improved and she did not continue with immunosuppressive therapy due to side-effects. She was then monitored in the renal clinic and had no further rheumatology follow-up until December 2017. On this occasion, she was referred to rheumatology from the neurology team in light of recurrent episodes of limb weakness and visual disturbances with an associated widespread livedoid rash. The neurological symptoms have been ongoing for two years, but appeared to be increasing in frequency. Three years prior to this, she developed Raynaud’s phenomenon affecting hands, feet, nose and ears. During the course of her illness, she was found to have a single low positive titre of cardiolipin IgM (14MPL kU/L) and also had one previously positive test for beta 2 glycoprotein 1, but subsequent testing for APS was negative. Subsequently, she presented as an acute admission to hospital, under the neurology team with headaches and transient dysphasia. An MRI was arranged and revealed a recent cortical infarct in the left parieto-occipital region, with multiple previous cortical infarcts. She was treated as a stroke secondary to possible vasculitis, in light of her younger age and initial MRI appearances. Further investigation with CT angiography did not reveal any changes to support vasculitis. Cerebrospinal fluid analysis obtained via lumbar puncture did not show any oligoclonal bands and had normal CSF protein and glucose values. Auto-immune antibody screen, including repeat antiphospholipid antibodies, ANA and ANCA and cryoglobulins were all normal. She has also had a trans-oesophageal echocardiography, which revealed a small patent foramen ovale. Skin opinion revealed that the rash was suggestive of livedo racemosa and organised a skin biopsy. The initial biopsy was unremarkable, but this will be repeated for confirmation. In light of her clinical presentation and suspicion of inflammatory disease, she was commenced on 60 mg of prednisolone. This was gradually reduced and she has now been initiated on azathioprine. She is also on antiplatelet therapy in the form of clopidogrel. Despite her immunosuppression, she continued to have symptoms of headache and fatigue but had no further focal neurological events.

**Discussion:** The patient has a background of IgA vasculitis which is inconsistent with the findings of livedo racemose and stroke. Therefore, she is likely to have an additional diagnosis for these symptoms. The patient had other risk factors for stroke in the form of a patent foramen ovale but did not have any evidence of venous thromboembolic disease or cardiac thrombi. Multiple strokes in a younger patient and livedoid phenomenon may also indicate antiphospholipid syndrome (APS). However, despite the transient detection of antiphospholipid antibodies at low tires in the past, this was considered insufficient for a diagnosis of APS. She also did not have any other features to suggest APS such as recurrent miscarriages or venous thromboembolic disease. The combination of multiple strokes and livedo racemosa is suggestive of Sneddon’s syndrome. Sneddon syndrome is thought to be associated with APS, but unlike APS is not always found to have positive antiphospholid antibodies. In the case of this patient, the initial skin biopsy was negative. The livedoid rash appeared to change very rapidly making identification of a suitable biopsy site difficult. Previous studies suggest multiple biopsies can increase sensitivity and this may be reflected in this case. Due to the relatively small number of patients with this condition, evidence for optimal management of these patients is limited. However there appears to be a role for anticoagulation/antiplatelet therapy and immunosuppression in more resistant cases. Literature suggests a role for steroid therapy and cyclophosphamide. Azathioprine has also been used in some cases with varying results. Although the patient did not achieve complete resolution of symptoms, the absence of any further ischaemic events may suggest a response to immunosuppressive therapy.

**Key Learning Points:** Sneddon's syndrome is a rare condition of multiple strokes and livedo racemosa. It is often associated with antiphospholipid antibodies and may be seen in anti-phospholipid syndrome. Skin biopsy can be useful in diagnoses, but multiple biopsies may be required to increase sensitivity. Treatment primarily involves antiplatelet therapy or anticoagulation. But some cases have been found to respond to immunosuppressive agents.

**Disclosure: H. Anver:** None. **S. Dubey:** None. **S. Banerjee:** None. **T. Potter:** None. **A. Short:** None. **A. Ilchyshyn:** None.

